# Household food insecurity, family size and their interactions on depression prevalence among teenage pregnant girls in Ghana, a population based cluster survey

**DOI:** 10.1186/s12905-023-02674-9

**Published:** 2023-10-06

**Authors:** Dominic Be-Ikuu Doglikuu, John Kwasi Annan, Stephen Asare, Hannah Yawson, Ohene Takyi, Francisca Akpene Dzidzornu, Henrietta Oye Koram, Edna Ayishetu Johnson

**Affiliations:** 1 Faculty of Health and Allied Sciences, Department of Nursing and Midwifery, Catholic University of Ghana, Sunyani-Fiapre, Bono Region, Ghana; 2 Department of Registered Public Health Nursing, Ministry of Health, Twifo Praso Nursing and Midwifery Training College, Central Region, Twifo Praso, Ghana; 3 Department of Registered Midwifery, Ministry of Health, Twifo Praso Nursing and Midwifery Training College, Central Region, Twifo Praso, Ghana; 4 College of Postgraduates studies, Department of Curriculum development, School of Nursing and Midwifery, University of Cape Coast, Central Region, Cape Coast, Ghana; 5 Department of Registered General Nursing, Ministry of Health, Twifo Praso Nursing and Midwifery Training College, Central Region, Twifo Praso, Ghana; 6 University of Energy and Natural Resources, Bono Region, Ghana; 7 College of Health and Allied Sciences, Department of Adult Health, School of Nursing and Midwifery, University of Cape Coast, Central Region, Cape Coast, Ghana; 8 Department of Registered General Nursing, Ministry of Health, Methodist Health Training Institute, Afosu-Campus, Afosu, Eastern Region Ghana

**Keywords:** Household food insecurity, Family size, Teenaged pregnancy, Depression, Ghana

## Abstract

**Background:**

Household food insecurity is the situation where individuals and families have limited/uncertain access to nutritionally adequate and safe foods for healthy living. Family size is the number of persons in the family. Household food insecurity and family size are risk factors for depression. However, their synergistic and/or multiplicative effect on depression is not well investigated. This study investigated/examined household food insecurity, family size and their interaction on depression prevalence among teenaged pregnant girls in Ghana.

**Methods:**

**Population based cluster survey was conducted among 225 teenaged pregnant girls** in 20-electoral areas at Twifo-Atti-Morkwa district in the central region of Ghana. Simple random sampling was used to recruit study participants and structured questionnaire used to collect demographic variables. Household Food Insecurity Access Scale (HFIAS) and Revised Children’s Anxiety and Depression Scale (RCADS-25) were used to collect data. **Multinomial logistic regression models were used to analyzed the data**.

**Results:**

Moderate and high depression prevalence reported among teenaged pregnant girls in Twifo-Atti-Morkwa district were 35.1(28.1–42.1) and 33.5 (26.5–40.5) respectively. Moderate family size (AOR = 1.08, 95%CI = 1.17–3.71) and large-family-size (AOR = 2.78, 95%CI = 3.98–10.19) were significant for depression. Moderate food insecurity (AOR = 0.12, 95%CI = 0.41 − 0.35) and high food insecurity (AOR = 0.27, 95%CI = 0.11–0.71) were significant for depression. Interaction between moderate food insecurity and moderate family size (AOR = 1.69, 95%CI = 2.79–17.51), interaction between high food insecurity and low family size (AOR = 1.24, 95%CI 1.57–11.41) and interaction between high food insecurity and large family size (AOR = 1.01, 95%CI = 1.72–14.57) were significant for depression among teenaged pregnant girls.

**Conclusion:**

There is moderate and high depression prevalence among teenaged pregnant girls in Twifo-Atti-Morkwa district. Interaction between household food insecurity and family size are the major predictors for depression among the teenaged girls in the district. We therefore recommend that public health officers should be up with health education campaigns in the district to create awareness on the depression prevalence among teenaged girls, and urge them to come out and seek support to prevent the catastrophic effect of depression.

## Introduction

Food security is the measure of food availability, and individuals’ ability to access it [1]. According to the United Nations’ Committee on World Food Security, food security is defined as the ability of all people, at all times, to have physical, social, and economic access to sufficient, safe, and nutritious food that meets their food preferences and dietary needs for active and healthy life [2]. This definition of food security incorporates a measure of resilience to future disruption or unavailability of critical food supply due to various risk factors including climate change, economic instability and wars. Individuals who are food secure do not live in hunger and/or fear of starvation. On the other hand food insecurity, is a situation where people have limited or uncertain access to nutritionally adequate and safe food or limited or uncertain ability to acquire acceptable food in socially acceptable manner [3, 4]. A person is food insecure when s/he lacks regular access to enough, safe and nutritious food for normal growth and development, and active and healthy life [4]. Food insecurity can be experienced at different levels of severity, and when a person is severely food insecure, s/he has run out of food and gone a day or more without eating. In other words, this person has experienced hunger [5].

Globally about 1.9 billion people experienced food insecurity in 2017, with the greatest numbers in Sub-Saharan Africa and South Asia [6]. About 9% of the world population i.e., 697 million people experience severe food insecurity [6]. The number of people unable to afford dissent or healthy diet around the world rose from 112 million in 2019 to almost 3.1 billion in 2020 [7]. In 2021, nearly 924 million people (11.7% of the global population) faced food insecurity at severe levels [7]. Currently close to 2.3 billion people in the world (29.3%) face moderate or severe food insecurity [7]. In Sub-Saharan Africa, about 30% of the population experienced severe food insecurity in 2020 [8]. In Ghana the prevalence of food insecurity is not different, it is reported that about 38.2% of Ghanaians experienced food insecurity [9]. Study shows that the major determinants of individuals vulnerabilities to food insecurity include clusters of risk factors such age of the household head, family size, availability of social safety net programs and death of household members [10]. Central to these clusters of risk factors in the Ghanaian context is the family. Families in Ghana play integral roles in individuals’ development, growth, health seeking behaviors, decision making processes and resources allocations. Therefore, disruption the family system in any form in Ghana could be counterproductive to health and wellbeing of the individuals’.

The word ‘’Family’’ originated from the Latin word ‘’familia,’’ which means a group of people related to one another either by consanguinity (birth) or affinity (marriage and other relationship) [11]. This definition, broadly count people as “family” even though they do not live together, but is related to one another biologically or through legal contracts. In contrast to this definition, other scholars defined family as a group of people not generally related to one another by blood, but share common attitudes, interests, or goals, and frequently live together [12]. In a more concise definition, family is a basic social unit comprising the parents and their dependent children living together in one household [13]. According to the most functional definitions, a family is any unit in which there exists: A sharing of resources, caring and supportive relationship with members [14].

Family size however, is the number of persons in the family [15]. There are two main types of family sizes: Large family size, and small family size. Large family size is the type where the number of members inside is large and thus, consists of the husband, wife, or wives, children, grandparents, uncles, aunts, cousins, nephews, nieces etc. [16]. Small family size on the other hand is the type of family that has fewer numbers, and traditionally refers to as the nuclear family system. This kind of family size consists of the husband, wife, or wives and children.

Analysing the definitions of food security and family size thereof, it is clear that these variables in their adverse forms could have high propensity of causing mental health problems including depression. In a large-scale family policy and food insecurity observational analysis in 142 countries, it has been reported that moderate or severe food insecurity was higher in households with large family size [17]. In another related study food insecurity was shown to be associated with depression, anxiety, and stress in the early days of the COVID-19 Pandemic [18]. Further, study in the US indicates that food insecurity is associated with 257% higher risk of anxiety (odds ratio: 3.57; 95% CI: 3.01 to 4.23) and 253% higher risk of depression (odds ratio: 3.53; 95% CI: 2.99 to 4.17) [19].

In the UK study shows that having three or more children in a family is associated with higher odds of lifetime depression [20]. In South Africa study also indicates that food insecurity and depressive symptomology increased during the COVID pandemic, with both men and women significantly reported rising depressive symptoms with different levels of food insecurity [21]. In Nigeria, being in a polygamous family (OR = 5.781, 95% CI, 3.253–24.371), and having a single parent (OR = 2.236, 95% CI, 0.869–11.786) were significantly associated with increased odds for depression among adolescents girls [22].

In Ghana, WHO reports indicates that approximately two million Ghanaians suffer from mental disorders including depression [23]. In the northern part of Ghana study indicates that about 20.1% of adolescent girls were classified as been likely depressed, and 70.3% of their households were food insecure, with 22.9% and 18.0% living in moderate and severe food insecure household respectively [24]. Another study in Savelugu Municipality also in the northern of Ghana indicates that teenaged girls in food insecure households were about three times more likely to be depressed than those in food secure households [25].

Although different studies have demonstrated significant associations between household food insecurity, and family size on depression prevalence among people in different countries, very little studies have been conducted to indicate how these variables could interact or act in tandem to influence depression and other mental health problems either in Ghana or elsewhere; thus, our study therefore seeks to fill this gap.

Individuals’ vulnerabilities to mental health problems increase with clusters of risk factors that can exist and/or act in tandem. Since these clusters of risk factors can exist and/or act in tandem to influence the outcomes of mental health problem including depression, it is important that researchers examine how these risk factors can independently, and synergistically act to influence depression prevalence among individuals in the population. Therefore our study seeks to assess how household food insecurity, family size and their interactions are related to depression prevalence among teenage pregnant girls in Ghana.

## Methodology

### Study area and population

This study was conducted at Twifo Atti-Morkwa district in the central region of Ghana. Twifo-Atti-Morkwa district is one of the districts in Ghana where small scale and illegal mining popularly known as ‘’Galamsey’’ have gained ground in Ghana, and destroyed major part of the green vegetation cover thus decreased agricultural activities, food production and supply. Also due to the illegal mining activities in the area couple with high level of poverty, many young girls in the district rampantly get pregnant and give birth thus leading to increased family sizes in their household. We conducted our study among teenaged pregnant girls because study indicates that more women in Ghana report depression prevalence during pregnancy [26], and factors associated with this include lower hopefulness, moderate and severe hunger, physical, and/or sexual intimate partner violence (IPV), and insufficient social support from female relatives [26]. **Study design**: The aim of our study was to investigate how household food insecurity, family size, and their interaction could be related to depression among teenaged pregnant girls in Ghana. To achieve this we decided to conduct a population based cluster survey among teenaged pregnant girls in the Twifo Atti-Morkwa district of central region of Ghana. We adopted this study design because Twifo Atti-Morkwa district is a small town in the central region in which small scale and illegal mining are on-going. By virtue of this the population tends to live in clusters with different population variance. We therefore adopted population based cluster survey to include more population variance in our study to accurately predict our outcome variable of interest.

#### Inclusion and exclusion criteria

Teenaged girls who were confirmed pregnant by a qualified medical doctor were included into our study. Also all teenaged girls who were seen physically pregnant by visual inspection and they also confirm that by showing in an antenatal attendant book given to them by healthcare workers were also included. On the other hand all teenaged girls who were not pregnant and those who were pregnant but were not medically and/or psychologically fit to answer our questionnaires were excluded.

### Sampling

#### Sample size determination

Single population proportion formula (n= (Z^2^ P (1-P))/e^2^) was used to determine the sample size of this study. In the stated formular, the letter ‘n’ denotes study sample size, ‘Z’ denotes the population standard normal distribution of 1.96 for 95% confidence interval, ‘P’ is the true population proportion or prevalence of depression among teenaged girls in the study area thus Twifo Atti-Morkwa district, and ’e’ is the standard error of (5%). Since we could not find any previous study in Twifo Atti-Morkwa district that reported prevalence of depression among teenaged girls, we used the national child depression prevalence rate of 13% in calculating our sample size [27]. Thu, substituting these values into the equation, the sample size n was calculated as n = 173. However, for the fear of non-response from participants’ and registration error by data collectors, a contingency sample of 30% was added to the estimated sample [28]. Considering this the final sample was increased to 225.

#### Sampling techniques

We used simple random sampling method in the WHO stepwise clusters survey to include 225 teenaged pregnant girls from the 28 electoral areas at Twifo Atti-Morkwa district into our study. The prevalence of household food insecurity and depression were estimated from these sub-group survey participants. In the WHO stepwise clusters survey, we employed multi-stage, clustered random sampling method, using the 2020 Ghana population and housing census data (61,743) of the area as the base line infomation. We used three-staged geographically clustered random sampling method to recruit our study participants. In the first stage, out of the 28 electoral areas, we used simple random sampling to choose 20 electoral areas. In the second stage, each selected electoral area is zoned into four and simple random sampling applied to select three zones. From each selected zone, houses and households were chosen to enter using systematic random sampling. Inside each house and in each household, where an adolescence pregnant girl was found, her consent was sought and interview granted, otherwise the process is repeated until an eligible participant is found and interviewed. These sampling processes were repeated in all the 20 selected electoral areas until we recruited and interviewed our sample size of 225 participants. This data collection process span through a period of 4 months (May to August, 2022).

### Data collection procedure

Research assistants were trained on how to collect the data using the structure questionnaire and the scales for measuring depression and food insecurity. These trained data collected were dispatched with the structured questionnaire to collect the data from the participants after the principal investigators have tested them and convinced that these research assistants were asking accurate questions that are consistent with the structured questionnaires and the various scales given to them. During the collection, demographic characteristics of participants thus, age, marital status, place of residence, educational background, parental occupation, family size, smocking, and alcohol intake status were collected using structured questionnaire. Household Food Insecurity Access Scale (HFIAS) developed by Food and Nutrition Technical Assistance (FANTA) Program of the U.S. Agency for International Development was used to measure participants household food insecurity [29]. The Household Food Insecurity Access Scale (HFIAS) is based on the idea that persons experiences of food insecurity (access) is caused by predictable reactions and responses that can be captured and quantified through survey and summarized in a scale [30]. The intent of the HFIAS is to provide a quantitative way for food security programs to easily measure the impact of their programs on the access component of household food insecurity. The HFIAS composed of a set of nine questions that is used to measure food insecurity in households in several countries, and appear to distinguish food insecure households from food secure ones across different cultural contexts [31].

We also used Revised Children’s Anxiety and Depression Scale (RCADS-25) to assess the prevalence of depression among teenaged pregnant girls in our study [32]. The Revised Children’s Anxiety and Depression Scale-25 (RCADS-25) is a 25-item scale that measures levels of anxiety (e.g. “I worry when I think I have done poorly at something”) and low mood (e.g. “I feel sad or empty”) [32]. All the items on the scale assess the frequency of symptoms and are rated on a 4-point Likert scale. This self-report scale is used among children and adolescents between the ages of 8 to 18 [33]. These questionnaires used in our study were pre-tested among 20 teenaged pregnant girls at Twifo Praso Policy Clinic in the same district to test for their internal validity and consistent before the main data collection. The results from the pretest indicate Cronbach’s alphas for RCADS scales 0.75, and that for Household Food Insecurity Access Scale (HFIAS) 0.94, indicating good internal consistency of the scales. The 20 teenaged pregnant girls who participated in our questionnaires pretest were excluded in the final study.

### Statistical analysis

The data collected from the field were captures into IBM SPSS version 20 (SPSS, Chicago, IL, USA). The captured data were cleaned and the normal distributions checked using Kolmogorov-Smirnov test. Descriptive statistics were used to describe participants’ demographic variables, One-way ANOVA with Post Hoc Bonferroni correction was used to compare significant mean differences across the three groups (low, moderate and high) of our dependent variable thus depression. Finally, multinomial logistic regression models were used to assess the association of household food insecurity, family size and their interactions for depression prevalence among the teenage pregnant girls in our study. The statistically significant of all the variables in our data was set at p-value ≤ 0.05.

#### Ethical considerations

This study protocol was approved by the Ghana Health Service Ethics Review Committee (GHS-ERC008/07/22). During the data collection, participants were assured of anonymity, participants’ names or anything that identified them was not collected. Participants were also told that their participation in the study was voluntary, and that they have the right to stop participating in this study at any time without having to give any reason or being forced to do so. Each participant was also asked to sign a written informed consent before participating in the study.

## Results

### Demographic characteristics family size and household food security

Participants’ demographic characteristics are presented in Table 1. About 43% of the study participants were aged between 10 and 16 years, close to 58% aged between 17 and 20 years, and about 2% was married. With regard to family size close to 95% live in large family size, and the rest lived in moderate and low family sizes.

**Table 1:** Participants Demographic characteristics

Variable	Frequency (%)
**Age category**	
10–16	85(42.5)
17–20	115(57.5)
**Marital status**	
married	3(1.5)
Single	197(98.5)
**Place of residence**	
Rural area	46(23.0)
Urban	150(75)
**Educational background**	
No formal education	12(6)
Primary	25(12.5)
Junior high	91(45.5)
Senior high	64(32)
Other form of education	8(4.0)
**Parental occupation**	
Health worker	1(0.5)
Trader	16(8.0)
Famer	3 (1.5)
Apprentice	1(0.5)
Other occupation	150(75)
**Family size**	
Small family size	4(2)
Moderate family size	7(3.5)
Large family size	189(94.5)
**Smocking status**	
Declined to answer	8(4.0)
Yes (Smock)	2(1)
No (Do not smock)	190(95)

***Small family size is defined as 1-3members in the household, moderate family size is defined as 4–6 members in the household, large family size is defined as 7 members and above in the household***

### Prevalence of food insecurity and depression

Participants’ household food insecurity and depression prevalence are presented in Table 2. Participants reported moderate and high household food insecurity as 35.1% and 38.4% respectively. Participants’ depression prevalence was reported as 35.1% and 33.5% for moderate and high depression respectively.

**Table 2:** Household Food Insecurity and Depression Prevalence among Pregnant Teenage Girls in Twifo Praso

Food Insecurity n (%)		Depression n (%)	
low	26.5 (20.5–32.5)	low	31.4 (25.4–37.4)
moderate	35.1 (28.1–42.1)	moderate	35.1(28.1–42.1)
high	38.4(31.4–42.5)	high	33.5 (26.5–40.5)

There was significant mean difference in household food insecurity and depression prevalence among participants (p-value ≤ 0.001) (Table 3).

**Table 3:** Comparison of Means Differences of Participants’ Demographic Characteristics, Household Food Insecurity with Depression

	Depression							
Variable	Low		Moderate		High			
	n	Mean (SD)	N	Mean (SD)	n	Mean (SD)	F-statistics (df1, df2)^**a**^	P-value^**b**^
Age (years)	60	1.867(0.650)	67	1.866(0.625)	64	1.953(0.6024)	0.411	0.663
Systolic blood pressure (mmHg)	60	107.550(7.489)	67	104.089(14.87)	64	110.031(10.718)	0.63	0.554
**Diastolic blood pressure(mmHg)**	**60**	**68.317(7.370)**	**67**	**66.8657(8.778)**	**64**	**68.5938(11.652)**	**4.369**	0.014*
Place of residence	60	1.867(0.700)	67	1.882(0.769)	64	1.750(0.436)	0.776	0.462
Family size	*60*	1.000(0.00)	67	1.045(0.208)	64	1.063(0.234)	1.816	0.166
Marital status	60	1.983(0.129)	67	2.000(0.000)	64	1.984(0.125)	0.542	0.582
Education level	60	3.400(0.061)	67	3.119(0.879)	64	3.109(1.086)	1.647	0.195
Smocking status	60	1.867(0.503)	67	1.940(0.295)	64	1.906(0.426)		
**Household food insecurity**	**58**	**2.434(0.728)**	**62**	**2.130(0.799)**	**59**	**1.78(0.767)**	**10.593**	0.0001**

^***a***^***One-way ANOVA***, ^***b***^Post-hoc analysis with Bonferroni correction that shows significant mean differences in: Diastolic blood pressure for low, moderate and high depression (P-value = 0.014), and Household food insecurity for low, moderate and high depression depression (P-value = 0.0001) *p-value > 0.001, **p-value ≤ 0.001

### Predictors of depression

The relation of household food insecurity, family size and their interaction for depression among teenage pregnant girls in Twifo Atti-Morkwa district is presented in Table 4. After adjusting for confounding variables (educational level, and parental occupation), moderate food insecurity (Adjusted Odd Ratio (AOR) = 0.12, 95% Confidence Interval (95%CI) = 0.41 − 0.35), and high food insecurity (AOR = 0.27, 95%CI = 0.11–0.71) were significant for depression. Moderate family size (AOR = 1.08, 95%CI = 1.17–3.71) and large family size (AOR = 2.78, 95%CI = 3.98–10.19) were significant for depression. The interaction between moderate food insecurity and moderate family size (AOR = 1.69, 95%CI = 2.79–17.51) and the interaction between high food insecurity and low family size (AOR = 1.24, 95%CI = 1.57–11.41) were significant for depression. Finally, interaction between high food insecurity and large family size (AOR = 1.01, 95%CI = 1.72–14.57) was significant for depression among teenaged pregnant girls.

**Table 4:** Association of Household Food Insecurity, Family size and their Interaction for Depression among Teenage Pregnant Girls Small family size is defined as 1-3members in the household, moderate family size is defined as 4–6 members in the household, large family size is defined as 7 members and above in the household. The model is adjusted for educational level and parental occupation *p-value > 0.001, **p-value ≤ 0.001

Variable		Moderate Depression COR (95%CI)	P-value	High Depression COR (95%CI)	P-value	Moderate Depression AOR (95%CI)	P-value	High Depression AOR (95%CI)	P-value
**Low family size**	Ref	1		1		1		1	
Moderate		**0.25(0.98 − 0.65)**	**0.004**	**1.03(1.16–3.54)**	**0.013**	**1.08(1.25–3.43)**	**0.004**	**1.08(1.17–3.71)**	0.013*
Large		**1.94(1.19–3.17)**	**0.008**	**5.61(3.24–9.69)**	**0.001**	**1.79(1.03–3.13)**	**0.040**	**2.78(3.98–10.19)**	0.001**
**Low food Insecurity**	**Ref**	**1**		**1**		**1**		**1**	
Moderate		**0.13(0.04–0.37)**	**0.000**	**0.30(0.12–0.79)**	**0.015**	**0.25(0.98 − 0.65)**	**0.043**	**0.12 (0.41 − 0.35)**	0.001**
High		**0.30 (0.12–0.78)**	**0.013**	**0.43(0.17–1.11)**	**0.082**	**0.33(0.13–0.84)**	**0.020**	**0.27(0.11–0.71)**	0.008*
Low Food Insec*Low family size	**Ref**	**1**		**1**		**1**		**1**	
Mod.Food Insec*Low family size		2.20(0.98–4.93)	0.055	1.72(0.66–4.48)	0.269	**2.40(1.05–5.47)**	**0.038**	1.87(0.70–4.99)	0.213
Mod. Food Insec*Mod. family size		3.63(1.54–8.57)	0.003	1.35(1.68–11.28)	0.002	1.98(0.80–4.91)	0.142	**1.69(2.79–17.51)**	0.001**
Mod. Food Insec*Large family size		2.47(1.07–5.67)	0.034	2.41(0.93–6.27)	0.070	**1.15(1.23–9.68)**	**0.019**	**1.61(3.73–24.96)**	0.001**
High Food insec *Low family size		1.77(0.81–4.81)	0.134	1.29(2.58–15.33)	0.0001	**1.22(1.63–9.45)**	**0.002**	**1.24(1.57–11.41)**	0.004*
High food insec*Mod. family size		1.15(1.26–9.47)	0.016	2.51(3.07–23.59)	0.0001	2.40 (0.90–6.44)	0.081	**2.06(1.09–5.97)**	0.030*
High Food insec*Large family size		1.75(0.74–4.14)	0.205	1.54(0.56–4.26)	0.404	**1.88(1.89–12.60)**	**0.001**	**1.01(1.72–14.57)**	0.003*

## Discussion

The aim of our study was to investigate how household food insecurity, family size, and their interaction relate to depression among teenaged pregnant girls in Ghana. At the end of the investigation we found that majority of teenaged pregnant girls reported modrate and high depression prevalence. There were also moderate and high prevalence of household food insecurity among these teenaged pregnant girls in the district.

The predictors or risk factors associated with the depression prevalence among the teenaged pregnant girls in the Twifo Atti-Morkwa district are household food insecurity; family size; and the interaction between household food insecurity and family size.

Depression prevalence has been reported among different sub-population in Ghana [34–37]. However, little evidence exists to show how different clusters of risk factors interact synergistically or multiplicatively to influence depression; thus, our study therefore seeks to breach this gap. In our study we found that moderate and large family sizes were related to depression prevalence among teenaged pregnant girls. This observation could be true because people living in large families may lack access to basic amenities including health care, cloths, shelter, and food. These lacks may produce stresses that could consequently produce early symptomatology of depression. As the teenaged girls struggle with coping, rumination and self-blame as a result of the stresses they received from life events, when not identified and managed timely, they may slip into overt depression. These findings in our study are consistent with findings among Nigerian adolescence [38]. In that study the authors reported that large family size in Nigeria was associated with higher odds of depression [38]. Study in South African among pregnant women living in low socio-economic setting also supported our findings. Result from that study found that pregnant women living in food insecure homes with three or more children have increased odds of suicidal behaviour and depression [39].

We found that moderate and high food insecurity was related to depression prevalence among teenaged pregnant girls in our study. This finding too could possibly be true because study show that presence of food insecurity is associated with mental ill-health, especially anxiety and depression among women [40]. Women who experience food insecurity feel uncertain with regard to whether food supplies will consistently remained accessible to them in the present and/or in the future, and this generates stress which consequently triggers anxiety and depression.

In our study we found that interaction between household food insecurity and family size was related to depression among adolescent/teenaged pregnant girls. When household food insecurity prevalence interacts with family size, we realized that adolescent/teenaged pregnant girls presented stronger odds of depression. These findings could also be true because both variables (household food insecurity and family size) are counterproductive to depression. When teenaged girls/pregnant girls live in large families with food insecurity, they share the problem and the different coping strategies their families go through to adapt. However, since these teenaged girls are not physically and mentally matured to become pregnant (teenaged pregnancy), and society also viewed their problem as socially undesirable or unacceptable, all these put together increased the risk of depression among these teenaged pregnant girls. As these risk factors already exist in the teenaged pregnant girl, when household food insecurity and large family size interact and add up, the situation worsened, and thus pushes the teenaged girl into more profound depression.

At the molecular level, research show that hormones and chemical responsible for depression include serotonin, dopamine, noradrenaline, and γ-aminobutyric acid (GABA) [41]. Adolescent girls living in large families most often suffer from three key components of food insecurity: -inadequate access to food, inadequate supply of food, and inappropriate utilization of food (e.g., inappropriate preparation of food) [42]. Per the explanation of food insecurity, people in moderate food insecurity might have to sacrifice other basic needs just as to be able to eat, and when they do eat, it might be whatever food that is readily available, which might not be nutritious. Adolescent girls in particular, who are exposed to food insecurity, often resort to meals skipping, and have strong desires for sweet as part of their copping mechanism or strategies which are not nutritionally healthy. When teenaged girls adopt to these poor eating habits and dietary patterns due to food insecurity over time, they developed many micronutrients deficiencies including essential amino acids such as tryptophan, tyrosine, phenylalanine, and methionine that are helpful in the production of the neurotransmitters; serotonin, dopamine, noradrenaline, and γ-aminobutyric acid (GABA)  which are essential for depression [43]. To this extent we can see the complex pathways through which household food insecurity and family size could cause hormonal imbalance leading to ill health among people.

Although we found positive associations between household food insecurity, family size and depression in our study, similar studies conducted elsewhere have shown much more positive association. For instance, study in South Africa reported that living in a polygamous family could result in as much as 478% odd of depression [21], while our study reported just 178% odd. This difference in magnitude of our results with others could probably occur due to differences in sampling procedure. While our study used multi-stage geographically clustered sampling method, the other used simple random sampling. The other reasons that could account for the difference could be due to differences in the sample population and sample size. In our study we sampled 225 teenaged pregnant adolescent girls’ while the other study sampled 540 in-school adolescents. Despite the differences in magnitude of our study results with others, we can conclude that household food insecurity and family size are strong predictors of depression among people.

### Strength and limitations

We conducted our study using a representative sample of adolescent girls in Twifo Attimorkwa district by following standard guidelines (WHO STEPS methodology) as prescribed by WHO in our population-based depression prevalence and risk factors assessment. This increases the generalizability of our study findings. Despite this strength, our study equally suffered certain limitations. Firstly, the individual risk factor assessment (household food insecurity) was assessed based on participants’ ability to recall, which could carry a certain degree of recall bias. There could also be an underestimation of some behavioral risk factors such as tobacco intake and alcohol consumption, due to socially desirable responses that tend to occur in interview-based surveys, also during pregnancy there are hormonal changes that could affect mood of pregnant women, this phenomenon could also lead to under or over estimation of our outcome variable (depression). However, the use of standard questionnaire, and trained investigators in the data collection techniques have helped us minimized some of these biases especially the recall and social desirability biases.

## Conclusion and recommendation

Despite the limitations stated above, this study is significant because it provides further evidence of how food insecurity and family size can interplay to contribute to the mental health crisis being experienced among Ghanaian population. The depression prevalence in our study is 35.1% and 33.5% for moderate and high depression respectively, and the clusters of risk factors associated with this among teenaged pregnant girls in the district are household food insecurity, family size, and the interaction of these two variables. Therefore, we recommend that food assistance programs such as the Ghana Government school feeding program in the district should include take home ration given to teenaged girls to curb the food insecurity problem among the vulnerable girls in the district. We also recommend that health care workers especially public health nurses should be up with their public health education campaign on family planning, and deliberately target young girls with the family planning commodities to increase their up takes among these teenaged girls in the district to prevent teenaged pregnancy. We also recommend that adults with food insecurity issues should be made to have access to mental health resources and reach out for help.

## Data Availability

The datasets used and/or analyzed during the current study is available from the corresponding author on reasonable request.
